# Development of Diamond and Silicon MEMS Sensor Arrays with Integrated Readout for Vapor Detection

**DOI:** 10.3390/s17061163

**Published:** 2017-05-24

**Authors:** Maira Possas-Abreu, Farbod Ghassemi, Lionel Rousseau, Emmanuel Scorsone, Emilie Descours, Gaelle Lissorgues

**Affiliations:** 1 ESYCOM, ESIEE-Paris, Cité Descartes BP99, 93162 Noisy-le-Grand, France; farbod.ghassemi@esiee.fr (F.G.); lionel.rousseau@esiee.fr (L.R.); gaelle.lissorgues@esiee.fr (G.L.); 2 CEA, LIST, Diamond Sensor Laboratory, 91191 Gif-sur-Yvette, France; emmanuel.scorsone@cea.fr; 3 ISIPCA, 34–36 Rue du Parc de Clagny, 78000 Versailles, France; edescours@isipca.fr

**Keywords:** microcantilevers, electronic nose, VOC discrimination, gas sensors, sensor arrays, synthetic diamond

## Abstract

This paper reports on the development of an autonomous instrument based on an array of eight resonant microcantilevers for vapor detection. The fabricated sensors are label-free devices, allowing chemical and biological functionalization. In this work, sensors based on an array of silicon and synthetic diamond microcantilevers are sensitized with polymeric films for the detection of analytes. The main advantage of the proposed system is that sensors can be easily changed for another application or for cleaning since the developed gas cell presents removable electrical connections. We report the successful application of our electronic nose approach to detect 12 volatile organic compounds. Moreover, the response pattern of the cantilever arrays is interpreted via principal component analysis (PCA) techniques in order to identify samples.

## 1. Introduction

A recent study revealed that humans can discriminate among more than a trillion different smells [1] and the mammalian nose remains the primary “apparatus” used in many applications to evaluate the smell of products. Despite the recent progress in research in the field, the mammalian olfactory system is complex and mechanisms of olfaction are still not fully understood [2]. Driven by the needs of odour detection for medical applications, environmental monitoring, security or food monitoring, the development of electronic noses has increased over the years [3,4,5,6,7,8,9]. These sensing technologies operate by mimicking the manner that mammalian noses proceed to discriminate odorant volatile compounds. The first study in this field [10] reported that a system aiming to mimic the mammalian olfactory system may be composed of two main elements: roughly tuned receptor cells, not selective toward specific odorant molecules and a system capable of performing parallel processing of the output signals. The processing may include qualitative analysis of sensor signal reports, by using, for example, pattern recognition techniques. Since then, various electronic noses have been developed based on different sensor technologies and different identification and classification methods [11].

Multi-gas detection using portable systems requires the use of sensors adapted for this specific application, especially in terms of sensitivity, response time and recovery time, selectivity, application to a wide range of gases, simplicity and convenience with respect to the use and replacement of sensors, and equally important, the sensor life-time. To meet this demand, a large number of technologies, based on optical, mechanical and electrical techniques, have been developed for chemical transduction.

Regarding the choice of these sensor techniques, solutions are distinguished by the type of sensitive layer and the principle of transduction. The most used for electronic nose applications are: semiconductor metal oxides gas sensors, conducting polymer sensors [12], Surface Acoustic Wave (SAW) sensors [13], Quartz Crystal Microbalance (QCM) [14] and optical fibre sensors [15]. Gas sensors differ in size, sensitivity, operating temperature, response and recovery times.

Electronic noses technologies offer a cheaper alternative to existing analytical instruments such as gas chromatography, mass spectrometry or ion mobility spectrometry [16]. They are supposed to be an alternative mobile or transportable and easy to use. In this context, the size and the number of sensors are important parameters, not only to obtain a smaller instrument but also to promote the use of smaller gas volumes, smaller detection surfaces and shorter detection times.

In the last two decades, advances in the micro-electromechanical systems (MEMS) field have promoted the development of miniaturized sensors that are able to transduce mechanical energy (e.g., gravitational potential energy) to electrical energy. The operation principle of MEMS sensors is that chemical, physical or biological stimuli can be transduced to mechanical stimuli and affect mechanical characteristics of the sensor structure in a manner that these changes can be measured by electrical or optical means. In this context, bio-chemical detection is possible by measuring mass or surface stress changes. In particular, microcantilevers, the simplest MEMS structures, offer the possibility of label-free biochemical detection with very high sensitivity [17]. The sensitivity of resonant microcantilevers is related to the dimensional scale of these devices.

In this perspective, a microcantilever-based electronic nose can offer highly desirable characteristics, including fast responses, height sensitivity and being able to accommodate a large number of sensors in a small volume. Moreover, they are suitable for mass production, taking advantage of micro-machining techniques and circuit integration.

In this work, we report the fabrication and the development of a silicon and synthetic diamond microcantilever array-based electronic nose. In the MEMS field, silicon is widely used. In fact, processing methods such as etching and photolithography have been thoroughly developed by the electronics industry, and have been easily adapted for MEMS production. As a consequence, the development of new techniques has not been favoured. Nonetheless, diamond is expected to be a very promising alternative in the micro-sensors field. In fact, diamond is a highly suitable material for the manufacture of resonant microcantilevers because of its exceptional mechanical and thermal properties, biocompatibility as well as excellent hardness and robustness. Polycrystalline diamond can be an excellent choice for the fabrication of resonant sensors due to its high elasticity modulus (in the order of $10^{3}$ GPa [18]). Moreover, because of its carbon nature, this material is convenient for stable grafting of a wide range of bio-receptors by covalent C–C binding [19].

The originality of the study reported in this paper is related to the development of a complete modular and autonomous system which is designed from sensors (silicon and diamond) to signal processing to be low noise, sensitive and easy-to-operate. In order to increase the sensitivity of our microcantilever sensors to volatile organic compounds detection, a variety of polymer coatings has been used to coat microcantilever surfaces. These sensors present mass resolution down to the ng range. Finally, we report the successful application of this electronic nose approach to discriminate some volatile organic compounds.

## 2. Material and Methods

### 2.1. Microcantilevers

**Figure 1:**
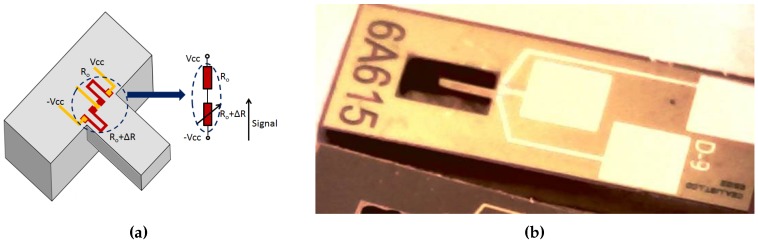
(**a**) Schematic of the placement of the piezoresistive gauges at the microcantilever anchorage and its equivalent circuit (**b**) A microscope picture of a silicon microcantilever with the three contact pads.

The MEMS sensor presented in this paper consists on an array of independent microcantilevers attached to a chip (2 cm × 5 cm) in which we can find electrical pads. We have fabricated silicon and polycrystalline diamond cantilevers using same geometry (same masks) in order to use in the functionalization step, a priori, silicon cantilevers for polymer coatings and diamond cantilevers for direct proteins binding. As diamond sensors have presented higher mass sensitivity than silicon ones, they have also been used with polymer coatings. The fabrication process of silicon and diamond cantilevers takes advantage of thin film technology and surface micro-machining techniques. In both cases, three metallic contact pads allow electrical connection to the integrated pair of poly-silicon strain gauges that serve as transducer elements. The piezoresistors in the cantilevers are connected to form a Wheatstone half-bridge circuit per chip as shown in Figure 1a. A silicon-based sensor is illustrated in Figure 1b.

**Figure 2:**
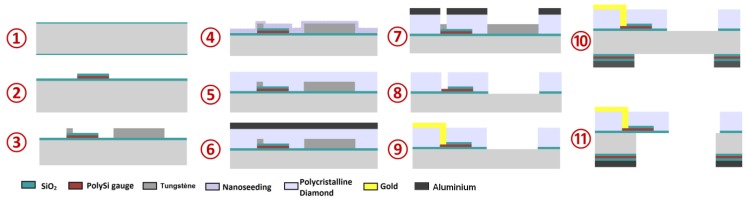
Polycrystalline diamond cantilever fabrication process. Fabrication steps start by structuring polysilicon gauges in order to place them at the bottom of the cantilever structure. Such a configuration is necessary to prevent the crystalline diamond layer from oxidising, which could occur during a subsequent step of depositing polysilicon. A layer of tungsten has been deposited to avoid diamond growth in the protected zones, since the diamond nanoparticles are spread on the whole wafer.

The fabrication of silicon cantilevers follows well-known techniques using a silicon-on-insulator wafer (SOI) as substrate [20]. In order to produce diamond microcantilevers, a novel polycrystalline diamond structuration method was developed and sensors were fabricated following a process previously described in [18]. Briefly, the fabrication process shown in Figure 2 starts with a four-inch single-side-polished silicon substrate. The wafer is thermally oxidized in order to create an electrical insulation layer (step 1). The strain gauges are created by sputter-deposition of polysilicon which is then patterned by etching (step 2). In order to prepare the wafer-to-diamond growth steps, a layer of tungsten has been deposited and patterned serving as an etch stop layer for a later etching step of synthetic diamond (step 3). In this work, diamond layer is structured by chemical vapor deposition (CVD). This technique consists of depositing carbon atoms over a substrate from methane gas using specific concentration, pressure and temperature conditions. Since polycrystalline diamond does not grow spontaneously on non-diamond materials, we seeded diamond nanoparticles over the substrate before proceeding to CVD steps. Nano-seeding of diamond is realized by incorporating a solution of diamond nanoparticles in Poly-vinyl alcohol (PVA) and by spin coating the solution over the substrate (step 4) using the process described in [21]. The wafer is then submitted to a Microwave Plasma Enhanced Chemical Vapor Deposition (MPECVD) reactor to synthesize diamond (step 5). After step 5, one can note that the diamond film is not homogeneous. The thickness varies over the wafer and on the zones protected by tungsten, the structure of diamond is fragile and thinner. In order to remove this layer, aluminium is sputter-deposited and structured using photolithography as a masking layer in opposition to the tungsten layer to generate the microcantilever shape and openings to access strain gauges (step 6). Subsequently, exposed diamond is removed by the Deep Reactive Ion Etching (DRIE) process (step 7). The aluminium mask and tungsten etch-stop layer are chemically removed (step 8). Immediately thereafter, the electrical contacts (chromium and gold) are structured by standard photolithography and etching techniques (step 9). Another aluminium masking layer is used to release the microcantilevers by performing DRIE etching of silicon on the wafer back side (steps 10 and 11). Diamond cantilevers have been designed to resonate at similar frequencies than silicon cantilevers of same length and width which means that diamond microcantilevers are thinner than the silicon ones.

**Figure 3:**
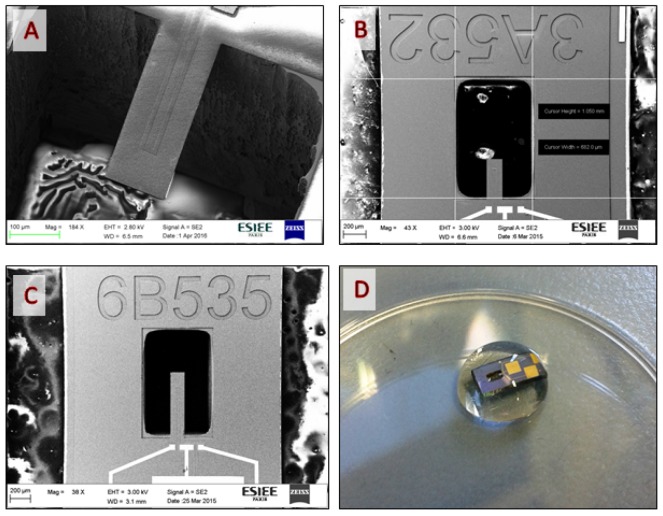
Pictures of some of manufactured devices: (**a**) SEM image of a beam Diamond L = 360 μm, (**b**) Same beam (**a**) with lower magnification (**c**) SEM image of a diamond beam L = 660 μm (**d**) Photography of a diamond cantilever during one of the rinsing steps.

Our sensors have been conceived to operate in dynamic mode to detect changes of the device’s mass due to adsorption of analytes on the microcantilever surface. For a microcantilever uniformly loaded on one side, as is the case of cantilevers coated with a sensitive layer, the mass change can be calculated from the measured resonance frequency shift using the following equation (model of harmonic oscillator):

(1) $$\Delta m=\frac{k}{4\pi^{2}}\left(\frac{1}{f_{2}^{2}}-\frac{1}{f_{1}^{2}}\right)$$

where Δm is the mass variation of the sensor, or the adsorbed mass, *k* is its spring constant, and $f_{2}$ and $f_{1}$ are the final and initial frequency, respectively. The equation is valid when coating layers does not significantly change the cantilever spring constant. In order to characterize our MEMS devices, resonance frequency measurements have been performed using a Micro Scanning Laser Doppler vibrometer (Polytec). Grain size and morphology of the fabricated cantilevers have been verified using scanning electron microscopy (SEM).The average size of the diamond grain is 1 μm. Regarding frequency profiles characterizations, sensor responses are comprised between 20 kHz and 150 kHz for all geometries. This range of values corresponds to cantilevers of different lengths (five geometries). The thickness of diamond films can also vary over the wafer, producing sensors with different resonance frequency. After fabrication and measurement tests, cantilevers were selected regarding their quality factor (greater or equal to 600) and mass sensitivity (in the range of hundreds of Hz/ng). Figure 3 shows some examples of SEM images of diamond microcantilevers of different geometries (A,B,C) and a photo of a diamond cantilever during one of the rising steps (D).

### 2.2. Microcantilever Functionalization

Silicon microcantilevers conceived to be used as chemical sensors must be adapted so that their surface acquires high affinity to the target analyte. Many types of coating layers can be used to increase cantilevers chemical sensitivity and selectivity. Noble metal coating layers have been used because they can provide surfaces which can be modified to bind biological or synthetic receptors [22,23]. These materials can also be used for chemical detections, particularly for gases such as hydrogen and mercury, for which we can use palladium and gold coatings respectively [24,25]. In the field of vapor detection, gas sensors are conceived to detect complex volatile organic compound (VOC) mixtures. Many studies have demonstrated that the use of polymeric materials in the form of thick films can increase the sensitivity of mechanical sensors to several volatile organic compounds [26,27,28]. Inorganic coatings such as zeolithes have also been used for VOC detection [29].

In terms of diamond chemical sensors, several possibilities can be envisaged to increase sensor sensitivity. Because of their carbon nature, diamond sensors are good candidates for stable grafting of a wide range of biological receptors via covalent C-–C binding [30,31,32,33]. In addition to present excellent chemical properties, diamond is well rated for sensors development due to its very high hardness and its inertness. As a bulk material, diamond sensors can also be used with other types of coatings as well as silicon sensors.

**Figure 4:**
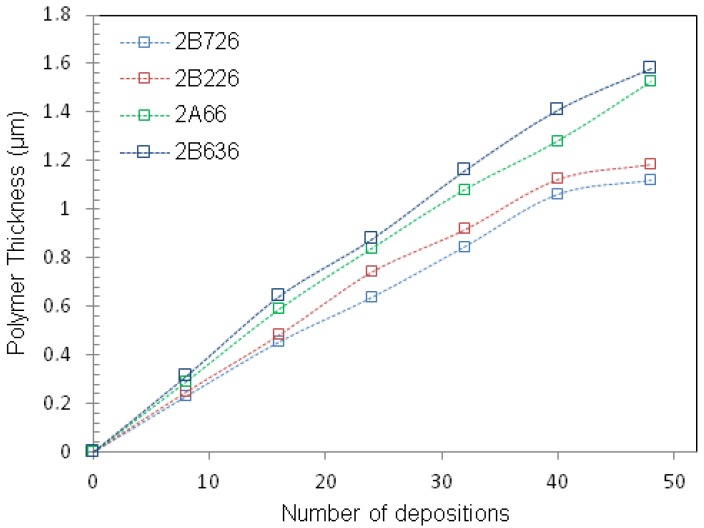
(**a**) Polymer film thickness as a function of the number of depositions for four microcantilevers of width = 140 μm and length = 260 μm (**b**) Schematic of the spray coating technique using a shadow mask to limit the area to be exposed over the microcantilever.

In this study, polymeric materials have been used as thin film coatings for silicon and diamond microcantilevers. Polymer solutions were deposited on diamond and silicon microcantilevers by a spray coating and in some cases, by spreading a droplet. A commercial airbrush (Evolution Silverline FPC, Harder & Steenbeck GMBH & CO., Norderstedt, Germany) was the equipment used for spray coating. The devices were cleaned prior to the polymer solution deposition (deionized water). Moreover, distance between sample and nozzle together with the pressure have been optimized to allow reproducible layer deposition and avoid the formation of droplets. Freshly coated microcantilevers were brought to 40 °C in an oven for 30 min in order to evaporate solvents. Film thickness was controlled by varying the concentration of the polymer solution and the number of depositions steps. Figure 4a presents the results for the characterization of the spray coating process. A shadow mask was fabricated to determine the area to be exposed (Figure 4b).

**Figure 5:**
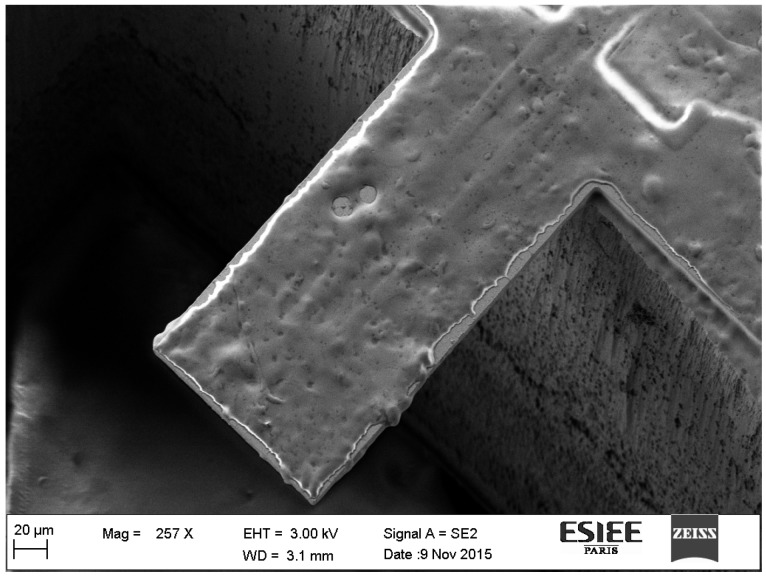
Silicon microcantilever coated with a film of polyepichlorohydrin (PECH) polymer by spray coating. The polymer solution was prepared by dissolving PECH on tetrahydrofuran (THF) at less than 5% of the mass of THF. Final polymer thickness = 2.42 μm.

Film thickness was measured using a mechanical profilometer. Because microcantilevers are flexible, it is not possible to measure polymer thickness on the microcantilever surface. Therefore, film thickness can be measured over the immobile part of the device thanks to the shadow mask corners. The validity of thickness measurements was confirmed by estimations of the deposit mass of polymer via the measurements of resonance frequency shifts of bare and coated microcantilevers. The thickness of the layer can be calculated from the estimated added mass, the density of the polymer and the area of the coated surface. Figure 5 presents a SEM image of a silicon microcantilever coated with poly(epichlorohydrin) (PECH) polymer.

**Figure 6:**
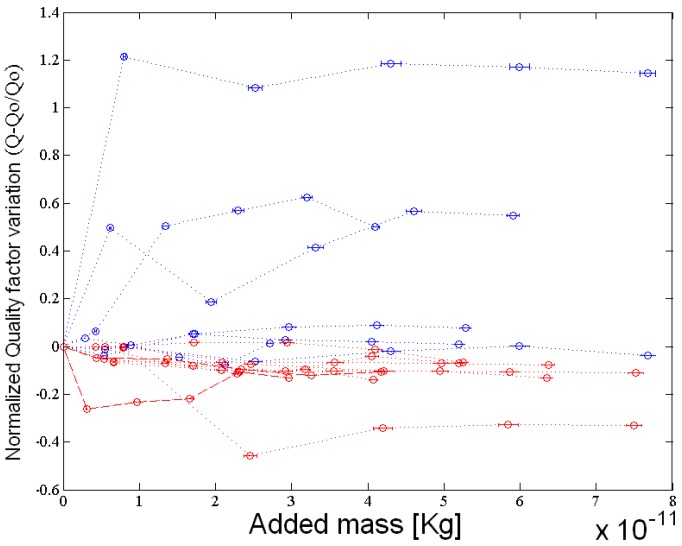
Relative changes in quality factor for diamond (**blue**) and silicon (**red**) for microcantilevers of different geometries.

The impact of coating on microcantilever sensors regarding energy losses depends on the geometry and the material of the sensor. We have observed that, for polymer thin layers (low added mass), the quality factor does not present significant changes for silicon cantilevers as can be observed in Figure 6. In the case of diamond sensors, the quality factor seems to increase when we add mass. In fact, this behaviour remains unexplained. One possible explanation is that as diamond cantilevers present a higher spring constant (due to the high elastic modulus), the added polysilicon acts like an added mass to a resonant system and does not degrade the equivalent elastic modulus. Thus, the quality factor of the systems may increase with increasing of mass.

### 2.3. Gas Cell and Electronic System

Detection of vapors using MEMS sensors relies on real-time measurements of cantilever deflection or cantilever resonance properties with high accuracy. Therefore, actuation and readout systems are a very important part of the development of resonant microcantilever-based sensors. The first aspect to consider is a practical aspect: microcantilevers must be placed inside a hermetically sealed gas cell which may also allow the direct actuation and read-out of the sensors. Another important characteristic of the cell is its volume size and geometry. In order to reduce time of detection, it is necessary to work with small volumes of gas. Internal geometry may also be optimized to avoid dead corners and provide a homogeneous gas flow.

**Figure 7:**
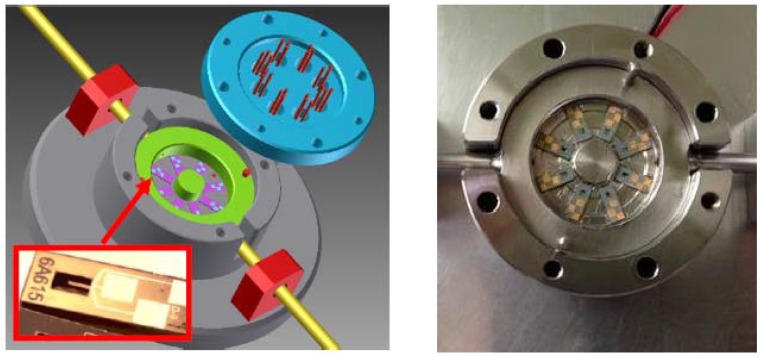
Three-dimensional drawing of the gas analysis cell showing the MEMS cantilever sensors placed inside (**left**) and a photo of the gas cell with eight diamond sensors placed inside (**right**). The internal volume of the cell is 1 cm^3^, small enough to ensure homogeneity of the gas inside. Furthermore, the main advantage of this gas cell is that it is easily adaptable to other applications, since it is possible to easily exchange one or more sensors in order to take account of the gases detected (patented).

In this context, a customized gas analysis cell has been conceived to accommodate up to eight sensors. The cell consists of a stainless steel chamber which presents eight cavities to accommodate sensors. A 2 mm rigid conduit is attached to the chamber serving as a gas inlet/outlet (Figure 7). Sensor chips are organized radially on a removable part (shown in green in Figure 7). A piezoelectric cell placed under the removable part is used to excite each microcantilever to its resonance frequency (first transverse mode). The design and volume of the chamber was optimized to create a homogeneous flow over all sensors and to avoid the dead corners using a minimum sample volume of gas. The cell is hermetically sealed and electrical contacts to the sensors are provided by using 24 spring-loaded pins (eight times three pads per cantilever). These pins are soldered to a PCB board screwed on the chamber cover. In this configuration, no wire-bounding is needed and sensors can be easily and individually changed for maintenance or for use in another application.

In order to properly interface our microcantilevers, we need to have an autonomous and stable electronic circuit that can detect very small changes in sensor resonance frequency (typically a few hertz in tens of kilohertz) over very long periods of time (minutes to hours). In recent years, the need to design superior instruments for electronic nose applications has drawn researchers attention to the development of new solutions [34,35]. In this context, we propose a low-noise and reconfigurable system as well as a dedicated human–machine interface. The major requirements in the design of this system are:

- Small dimensions: for electronic nose applications, it is not possible to use a measuring instrument such as gain-phase analyzers;
- A human–machine interface: an interface is necessary for the visualization of the data in real time and for the intervention on the measurement configurations;
- An analog processing interface to detect the responses of the sensors with sufficient resolution;
- A memory to store a certain quantity of measurements and calibration data of the sensors;
- An autonomous architecture: calibration and diagnostics executed autonomously, without user intervention;
- Communication interfaces: to communicate with other modules (pump control system, human–machine interface, etc).

With regard to the operation principle, in dynamic mode, the electronic system can operate following two methods of frequency shift detection: oscillator configuration or frequency sweep mode. In this work, the variations caused by dispersions during sensors manufacturing and the fact that we use an array of sensors make the sweep mode the best choice since it allows modularity and is easy to implement. In this operation mode, a sine-sweep signal is used to excite sensors near their resonance frequency while measuring sensors’ response for each frequency. The resonance frequency (maximum amplitude response) is recorded while sensors are submitted to a reference gas in order to generate a baseline. As the sensor response is continuously monitored, when gas samples are sent to the sensors, shifts on the frequency response can be detected.

**Figure 8:**
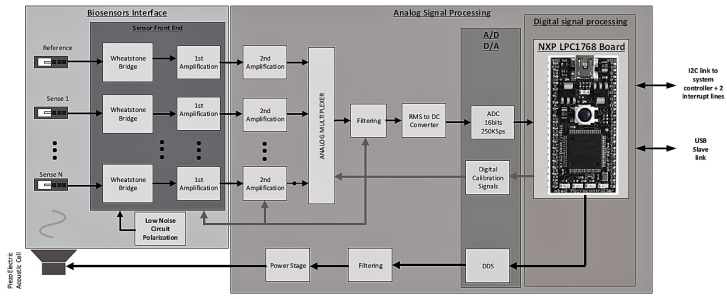
Functional architecture of the electronic processing circuit. The left part shows the biosensor interface corresponding to the board in Figure 9 (left). The analog signal processing circuit (right) corresponds to the board in Figure 9 (right).

**Figure 9:**
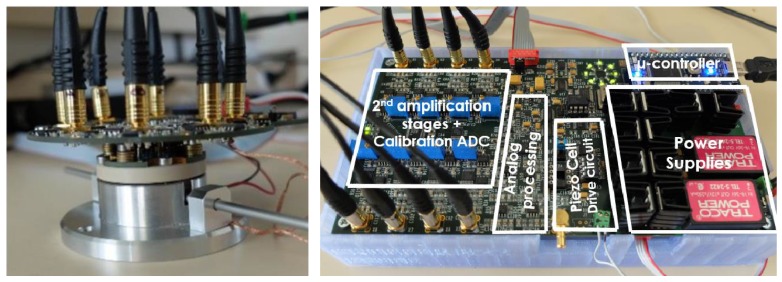
Gas analysis cell and electronic system—On the left: Bio-sensors interface circuit. On the right: Analog signal processing circuit.

Figure 8 provides a functional diagram of the electronic signal processing architecture. The left hand-side of the figure shows the interface circuit to the cantilever sensors. The piezoresistive gauges integrated in the microcantilevers allow an electronic reading of the resonance frequency. To obtain a compensation for the effects of temperature, another gauge is integrated in the substrate (fixed part) and is used as a reference gauge. Under the operating conditions used, our microcantilevers can vibrate at amplitudes of the order of a few tens of nanometers to a few hundred nanometers. For small oscillations, it is preferable to work in Wheatstone bridge configuration. This front-end interface allows the differential reading between the voltages of each half bridge. Low noise reference voltages have been used in order to maximize the signal-to-noise ratio. The pre-amplification stage is based on eight fixed gain instrumentation amplifiers (Analog Devices - AD8428) connected to the Wheatstone bridge. In order to balance each Wheatstone bridge and optimize the dynamic range of the output signal, digitally controlled potentiometers are connected in series with sensors resistances. In order to optimize the signal-to-noise ratio, this analog front-end is mounted straight on top of the gas cell as shown in Figure 9.

The second part (Figure 8, right) of the electronic system starts with a second amplification stage (Analog Devices—AD8429) with digitally programmable gain. Sensor signals are then time-multiplexed in order to use a single chain circuit. The signal is then treated through a digitally programmable low-pass filter (Linear Technology—LTC1565-31) which presents a cut-off frequency ranging from 10 kHz up to 150 kHz. To detect the maximum amplitude (related to the resonance frequency), the Root Mean Square (RMS) value of the filtered signal is computed using a delta-sigma RMS-to-DC converter (Linear Technology—LTC1968). The amplitude information is converted from analog to digital and sent to an embedded microcontroller (NXP LPC1768) which correlates each sensor amplitude response to an excitation frequency and builds up the eight corresponding frequency profiles. The sensors actuation signal sent to the piezo-electrical cell is generated through a Direct Digital Synthesizer (DDS—Analog Devices—AD5932). The sensors interface and processing board have been built apart in order to simplify mechanical assembly and reduce noise injection on the sensors interface board.

**Figure 10:**
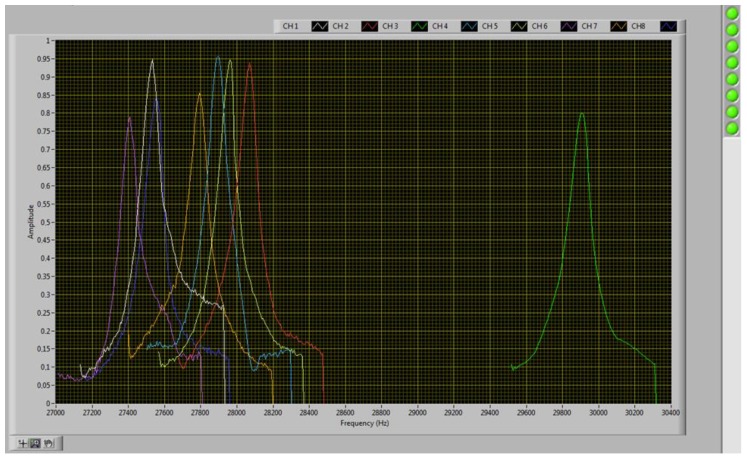
A picture of one of the screens of the Labview interface showing the real-time measurements of eight resonant microcantilevers. The curves are the frequency response profile of each sensor.

A dedicated LABVIEW application was developed to ensure communication between the micro-controller and the user. Figure 10 presents a picture of the LABVIEW interface showing the real-time measurement of the frequency response profile of eight cantilevers. This interface also allows the control of filters’ cut-off frequency and gain and the set-up of the frequency step and frequency span.

## 3. Experimental

### 3.1. Sensor Preparations

Eight cantilever sensors were functionalized by coating individual microcantilevers with six different polymer layers using the techniques presented in the previous section. Two diamond cantilevers have not been coated, but their surfaces were treated in order to change their hydrophobic properties. After the functionalization steps, we verified the mass changes and thickness of each cantilever in order to estimate the minimum concentration of volatile organic compounds to be used in this experiment. Table 1 summarizes the cantilever coatings used for this experiment.

### 3.2. Measurement Set-Up

**Figure 11:**
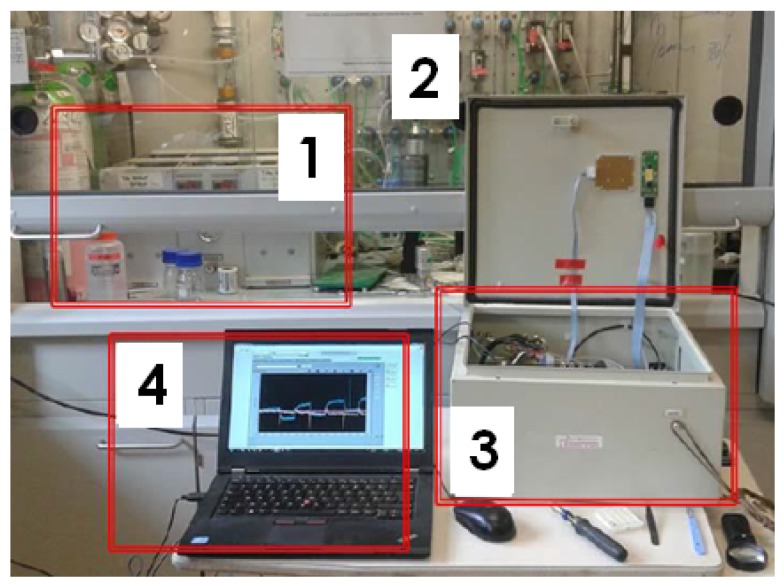
Picture of detection test set-up—1: volatile organic compound (VOC) generator; 2: photon ionization detector (PID) for real-time calibration; 3: the system under test; 4: PC to read-out of detection results.

The eight microcantilevers are placed on the gas analysis chamber in which gases and vapors can be introduced. The exposure to different VOCs was carried out by using a gas generator calibrated using a photon ionization detector (PID). Analytes in liquid phase are placed in a temperature controlled enclosure and headspace above the sample is carried in a stream of dry nitrogen gas to the measurement chamber via flow controllers and pumps. Detection set-up is completely independent on the system as can be seen in Figure 11.

Before using the sensors for the detection of vapors, we first checked the possible variations of original resonance frequency that are not related to the detection of analytes. Microcantilevers (bare or coated) are sensitive to pressure, temperature and humidity. We ensure the validity of the experiment by controlling the temperature and setting a constant flow of nitrogen at 0% RH.

Test conditions may also vary depending on the acquisition configurations of the electronic system. Indeed, the number of points (sampling step) and the frequency span of measurement are configured for each case so as to optimize the response time without deteriorating the accuracy of the measurement. This consists of reducing the frequency band to the minimum possible and also the sampling step in order to keep the maximum number of points focused on the resonance peak.

## 4. Results

**Figure 12:**
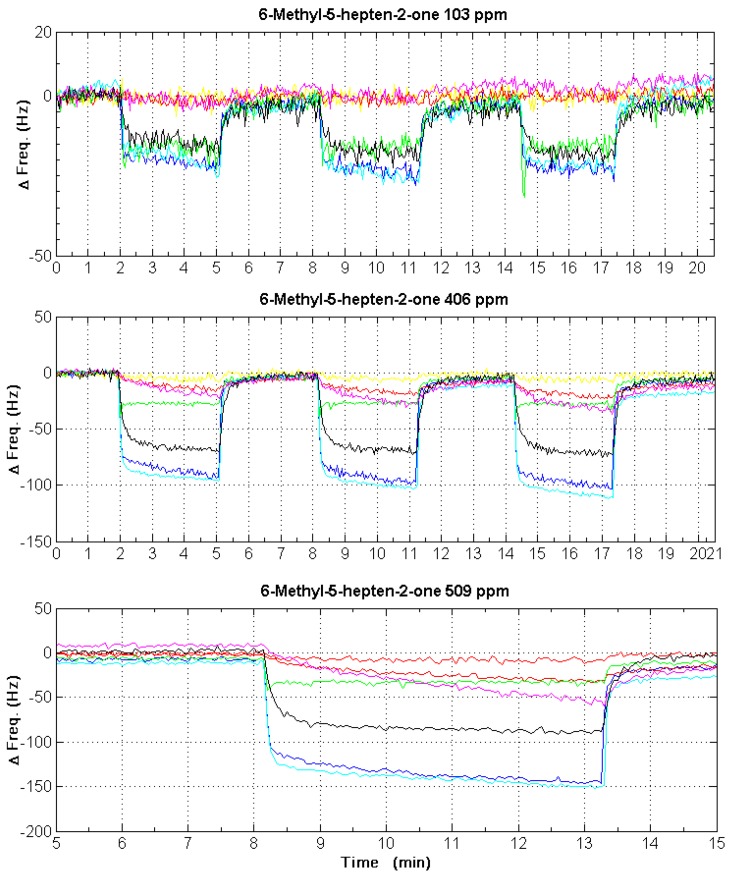
Cantilever responses for 6-Methyl-5-hepten-2-one at 103 ppm, 406 ppm and 509 ppm. Each concentration was generated many times in order to verify the repeatability and stability of our sensor array. Each colour in the graph represents the response of one sensor.

In this experiment, the detection of vapors is achieved by the diffusion of the analyte into the polymer layer and also by surface interaction with diamond sensors (bare cantilevers). As the operation mode for this application is the dynamic mode, changes in the polymer layers lead to an increase of sensor total mass, resulting in a negative frequency shift. Shifts of resonance frequency of each microcantilever are specific to the interaction between vapor molecules and the polymer. When we change to a flow of pure nitrogen in the same temperature and flow rate conditions, the trapped analyte starts to diffuse out of the sensitive layer, back to the environment, causing a decrease of mass and a positive resonant frequency shift. Figure 12 presents some examples of cantilever-array responses in dynamic mode. Considering the frequency shift as the system output signal, the signal-to-noise ratio will depend on the sensitive layer and the substance to be detected. However, in any case, the estimated noise level (fluctuation of the output signal when the system receives reference gas) is 4 Hz.

For each vapor tested, we have generated different concentrations in order to verify the linearity of the microcantilever sensors. Each concentration was also repeated at least three times in order to ensure that the response is reversible as shown in Figure 12.

**Figure 13:**
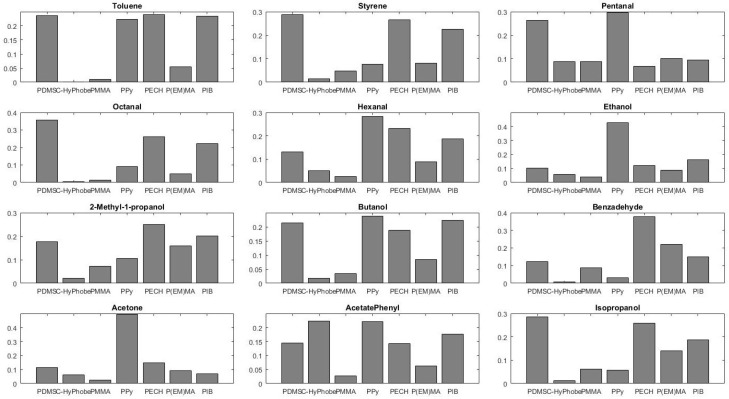
Relative response patterns for 12 vapors on seven microcantilevers with different coatings. The eighth one was used as reference and is not shown. The highest sensitivity was estimated for the couple “Ppy-Phenyl Acetate” and is about 1.62 Hz/ppm. For this case, LOD is estimated at 7.5 ppm.

This procedure was repeated for 13 substances : Toluene; Styrene; Pentanal; Octanal; Hexanal; Ethanol; 2-Methyl-1-propanol; Butanol; Benzadehyde; Acetone; 6-Methyl-5-hepten-2-one; Phenyl acetate; Isopropanol. Each vapor was generated at 500 ppm and sensor response (resonance frequency shift) was measured. Relative response patterns for 12 of 13 vapors are shown in Figure 13. The best sensitivity was estimated for the couple “Ppy-Phenyl Acetate” and is about 1.62 Hz/ppm. Considering the noise level of 4 Hz, the limit-of-detection in this case is 7.5 ppm. The relative response of a sensor (coating) is equal to the frequency shift of the sensor divided by the sum of the shifts for all eight sensor coatings. The sum of these scaled responses is the unity for each vapor, which facilitates comparisons. As sensor 2 was a diamond cantilever without coating, it was used as reference. Therefore, the response of this sensor is close to 0 Hz and was not represented in Figure 13. As we can observe, patterns of vapors are all different from each other. From Figure 13, we have an indication of the discriminating capability of our sensor array for this group of solvents. Some similarities can be found among the patterns from the same chemical class. For example, response patterns for ethanol and acetone are similar. One can also notice the complexity of the problem of recognizing and discriminating among more than a few vapors.

**Figure 14:**
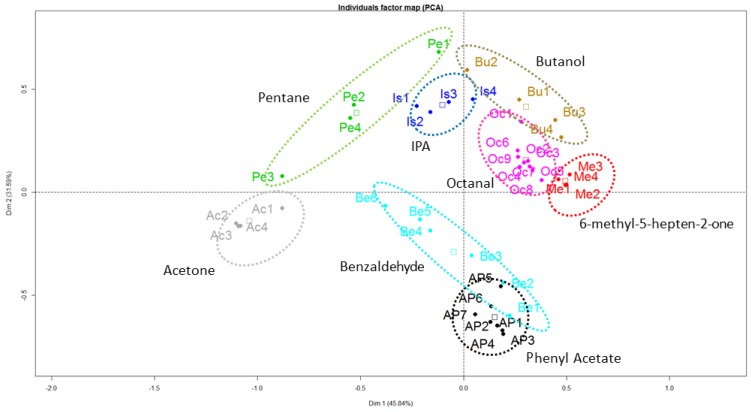
Principal Component Analysis (PCA) performed on results of some detected VOCs in a two-dimensional principal component space—Components 1 and 2. PCA shows good discrimination between species.

In order to identify the tested samples and evaluate the selectivity of the system, cantilever-array responses of different concentrations have been used to generate a set of “time-shift” vectors that corresponds to the dynamic development of the detection curves for each analyte. The data set was evaluated using principal component analysis (PCA) techniques, allowing to extract the most dominant deviations in the responses for the various sample vapors. In order to reduce the complexity of the analysis, we have only kept eight analytes, the best represented on the PCA first plane. As shown in Figure 14, each VOC is comprised of a cluster without any overlap. This result demonstrates the ability of the system to discriminate a large number of VOCs in dynamic mode.

## 5. Conclusions

In this work, we have reported the development of a system for vapor detection. Firstly, development results address the fabrication and characterization of silicon and diamond microcantilever-based sensors for VOC detection. In this study, microcantilevers were coated with different polymer layers in order to improve the sensitivity of the sensor array. A complete three-element electronic nose system (sensors, electronics and data treatment) was developed and we have demonstrated that an array of resonant micromechanical cantilevers can be used as chemical sensors for electronic noses. The system developed is completely autonomous and modular. One of the most important results of the present work is the development of a gas analysis cell (patented) able to hold up to eight sensors which are exposed to a homogeneous gas flow while providing electrical read-out of sensors. Moreover, the gas cell also provides an easy and reliable actuation method employing a piezo-electrical cell. A dedicated electronic read-out circuit has been conceived in two different boards which provides low-noise detection of sensor output signals and accurately tracks frequency shifts. Sensor frequency profiles are updated to the user interface every second, which allows detections to be followed in real-time, during field operation. The system was used to detect 13 volatile organic compounds in the range of hundreds of ppm and PCA techniques were applied to identify samples. In conclusion, we have developed a microcantilever sensor array-based system that exhibits significant potential as a tool for vapor analysis. Improvements in the sensor quality factor, sensitivity and specificity of sensitive layers may be considered in future work in order to improve the identification and separation of samples. 

## Figures and Tables

**Table 1:** Cantilever coatings, surface treatment and resonance properties.

Cantilever	Material	Sensitive Layer (Solvent > 90%)		Resonant Frequency [Hz]
1	Silicon	PDMS	Polydimethylsiloxane	113,954
2	Diamond	Hydrophile treatment	-	28,026
3	Diamond	Hydrophobic treatment	-	31,549
4	Silicon	PMMA	Polymethylmethacrylate	127,136
5	Silicon	PAC	Poly(acetylene)	59,769
6	Silicon	PECH	Polyepichlorohydrin	126,627
7	Silicon	P(EM)MA	Poly(ethylene-co-methyl methacrylate)	124,236
8	Silicon	PIB	Polyisobutylene	125,388
